# On Fusogenicity of Positively Charged Phased-Separated Lipid Vesicles: Experiments and Computational Simulations

**DOI:** 10.3390/biom13101473

**Published:** 2023-09-30

**Authors:** Yifei Wang, Yerbol Palzhanov, Dang T. Dang, Annalisa Quaini, Maxim Olshanskii, Sheereen Majd

**Affiliations:** 1 Department of Biomedical Engineering, University of Houston, 3551 Cullen Blvd, Houston, TX 77204, USA; ywang147@uh.edu (Y.W.);; 2 Department of Mathematics, University of Houston, 3551 Cullen Blvd, Houston, TX 77204, USA; ypalzhanov@uh.edu (Y.P.); maolshanskiy@uh.edu (M.O.)

**Keywords:** membrane phase separation, fusogenic liposomes, cationic lipids, fluorescence microscopy, computational modeling

## Abstract

This paper studies the fusogenicity of cationic liposomes in relation to their surface distribution of cationic lipids and utilizes membrane phase separation to control this surface distribution. It is found that concentrating the cationic lipids into small surface patches on liposomes, through phase-separation, can enhance liposome’s fusogenicity. Further concentrating these lipids into smaller patches on the surface of liposomes led to an increased level of fusogenicity. These experimental findings are supported by numerical simulations using a mathematical model for phase-separated charged liposomes. Findings of this study may be used for design and development of highly fusogenic liposomes with minimal level of toxicity.

## 1. Introduction

Nano-scale liposomes have proven to be highly effective and versatile drug delivery vehicles as they rely on two mechanisms for cellular uptake: endocytosis [1,2,3] and membrane fusion [4,5,6]. Membrane fusion, which entails the integration of two different membranes, is particularly appealing for delivery of macromolecules because through this mechanism liposomes deliver their encapsulated cargo directly into the cytoplasm. Liposomes that contain cationic lipids [6,7], such as 1,2-dioleoyl-3-trimethylammonium-propane (DOTAP), are known for their high fusogenicity [8]. Cationic lipids, with their conical shape and cationic headgroup, are critical for fusion [9,10,11]. While these lipids are typically non-toxic at lower concentrations, concerns arise regarding their toxicity when used at higher concentrations, attributed to their tetraalkylammonium moiety [12]. Therefore, designing delivery liposomes that offer both high fusogenicity and low toxicity is a challenge. This challenge may be overcome by controlling the surface density of cationic DOTAP on the surface of liposomes using the membrane phase separation phenomenon.

Phase separation is a fundamental process that occurs in multicomponent lipid membranes with substantial unfavorable interactions among their lipid components [13]. In such membranes, segregation of lipids based on their favorable interactions leads to phase separation. This phenomenon regulates molecular organization in membranes and thus can be used to control the surface density of the membrane’s components. The ternary mixture of DOPC:DPPC:Chol is an example of a phase-separating composition that can, for instance, form a tightly-packed liquid ordered ($L_{o}$) phase and a loosely-packed liquid disordered ($L_{d}$) phase at certain molar ratios. We previously combined experiments and modeling to investigate the phase behavior in this lipid mixture [14,15]. Here, we aim to explore the use of phase-separation in a DOTAP:DOPC:DPPC:Chol mixture to modulate the surface density of DOTAP on liposomes and hence their fusogenicity.

**Figure 1:**
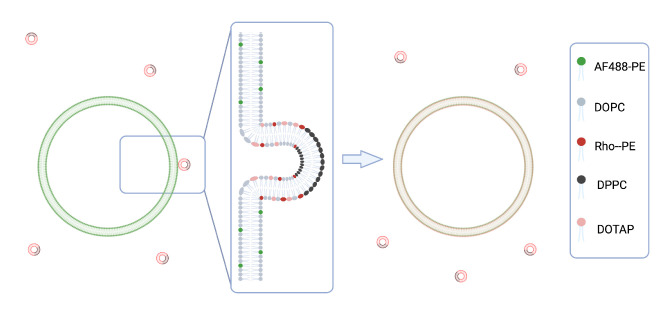
Schematic illustration of the phase-separated cationic SUVs (labeled with red-fluorescent lipids) fusing in to GUVs (labeled with green-fluorescent lipids).

We hypothesize that concentrating DOTAP into small patches on the liposome’s surface, through phase separation, can enhance the liposome’s fusogenicity without the need for high DOTAP concentrations. We further postulate that liposomes with the smallest patch area (i.e., the highest local density of DOTAP when the amount of DOTAP is kept fixed) would exhibit the highest level of fusogenicity into target membranes when compared to other liposomes with similar DOTAP content. To test these hypotheses, we examine the fusogenicity of nano-scale liposomes (referred to as small unilamellar vesicles—SUVs) of three different phase-separating compositions containing DOTAP (referred to as patchy liposomes—PAT) into micron-sized liposomes (referred to as giant unilamellar vesicles—GUVs) as model target membranes. Fluorescence microscopy was used as a tool to assess the level of SUV fusogenicity. To enable fluorescence microscopy as a gauge tool, different fluorescent lipids, Rho-PE and AF488-PE, were added to SUV and GUV membranes, respectively. The setup is schematically illustrated in Figure 1.

To complement our experimental studies, we further advanced the computational platform developed for [14,15]. Specifically, we included the electrostatic interaction between SUVs and GUVs into the surface Navier–Stokes–Cahn–Hilliard (NSCH) phase-field model, which accounts for phase behavior, domain formation, and membrane fluidity in ternary membrane compositions. In [15], we validated the continuum-based NSCH model without electrostatic interaction against experimental data and showed that our model predicts membrane phase behavior in a reliable and quantitative manner in the absence of DOTAP. The extension of the model presented in this paper enables predictions when DOTAP is present. This is a crucial step towards efficient computer-aided design of liposomes that use cationic lipids for high fusogenicity and membrane phase-separation for limited toxicity.

## 2. Materials and Methods

### 2.1. Experimental Approach

#### 2.1.1. Materials

Lipids 1,2-dioleoyl-sn-glycero-3-phosphocholine (DOPC), 1,2-dipalmitoyl-sn-glycero-3-phosphocholine (DPPC), 1,2-dioleoyl-3-trimethylammonium-propane (DOTAP), 1,2-dipalmitoyl-sn-glycero-3-phosphoethanolamine-N- (lissamine rhodamine B sulfonyl) (Rho-PE) and 1,2-dioleoyl-sn-glycero-3-phosphoethanolamine-N-(TopFluor^®^ AF488) (AF488-PE) were from Avanti Polar Lipids (Alabaster, AL, USA). We purchased the sucrose from Avantor (Radnor Township, PA, USA). Cholesterol was purchased from Sigma-Aldrich (Saint Louis, MO, USA) and chloroform from Omnipure (Caldwell, ID, USA). All lipid stock solutions were prepared in chloroform. Indium tin oxide (ITO)-coated glasses and microscope glass slides were from Thermo Fisher Scientific (Waltham, MA, USA) and coverslips were bought from Corning (Corning, NY, USA). ITO plates were cleaned with chloroform, ethanol and DI water before use. Microscope slides and coverslips were cleaned with ethanol and DI water.

#### 2.1.2. Preparation and Characterization of SUVs for Fusion Experiments

SUVs used for fusion experiments were formed by dehydration–rehydration followed by extrusion. In brief, a solution of the desired lipid mixture including the fluorescent-lipid, Rho-PE, in chloroform was prepared. Solution was placed in 10 mL round flask and dried under vacuum using a rotary evaporator (Hei-Vap, Heidolph, Germany) for 2 h. The produced thin lipid film was then hydrated using diluted (235 mM) PBS at a final lipid concentration of 3 mM, and then extruded through polycarbonate membranes with 100 nm pores (Cytiva, Marlborough, MA, USA) for 25 times. Both rehydration and extrusion were done at room temperature for homogenous SUVs and at 60 °C for phase-separating SUVs. Fusion experiments applied both homogenous and phase-separating SUVs. For the phase-separating SUVs, lipid composition DOPC:DPPC:Chol with three different molar ratios were selected (see Table 1), in which DOPC was partially replaced with DOTAP. We included Rho-PE to enable fluorescence microscopy.

A Malvern Zetasizer machine (Nano-ZS, Malvern Instruments, Malvern, UK) was used to characterize the SUVs for size distribution (via dynamic light scattering) and zeta potential (via laser Doppler electrophoresis).

#### 2.1.3. Preparation of GUVs

We employed a modified version of electroformation [14,16] to form GUVs. To this end, ∼ 35 μL of an aqueous dispersion of SUVs (in DI water) was deposited as small droplets onto two ITO plates. The droplets were left overnight to dry. Subsequently, a thin PDMS frame with integrated tubing was assembled between the ITO plates to create a chamber. To rehydrate the dried lipids, a solution of 235 mM sucrose was slowly injected into the chamber. The device was then placed in a 60 °C oven to exceed the highest lipid melting temperature in the mixture (in this case, DPPC with a melting temperature of 41.2 °C) for phase separated liposome or room temperature for DOPC GUVs.

To induce vesicle formation, an AC electrical field was applied using a function waveform generator (4055, BK Precision, Yorba Linda, CA, USA). The frequency was set at 50 Hz, and the electric field was gradually increased to 2 Vpp at a rate of 100 mV min^−1^. This field was kept for 3 h. Once the vesicles were formed, the frequency was reduced to 1 Hz for 30 min for GUVs detachment.

The SUVs used in the electroformation process were prepared using dehydration–rehydration followed by tip sonication. Note that while both extrusion and tip-sonication, post dehydration–rehydration procedure, can produce SUVs, the use of extrusion provides a better control over the SUVs’ size distribution and was thus selected for the preparation of SUVs in fusion experiments. In short, in a 5 mL pearl-shaped flask, DOPC and fluorescent lipid, AF488-PE, was mixed with chloroform. The details of this portion of the procedure are described in Section 2.1.2. Next, the size of the SUVs was reduced through tip sonication using a 55-Watt Sonicator Q55 (Qsonica, Newtown, CT, USA). The sonication process involved 30 s of resting followed by 1 min of sonication at 10 Hz. This sonication–rest cycle was repeated 20 times to obtain a clear solution of SUVs.

#### 2.1.4. Imaging and Analysis

For fusion experiments, SUVs of specified compositions and GUVs of DOPC were mixed at 1:1 molar ratio in microtubes and incubated at 37 °C for 10 min. The sample was then collected and placed on a clean microscope glass slide. Double-sided tape was used between the glass slide and a coverslip to create a chamber for imaging. All the images were acquired using Zeiss LSM 800 confocal laser scanning microscope (Zeiss, Germany). Confocal images were obtained using 63× oil objective with NA of 1.40 using 561 nm and 488 nm wavelength lasers. Over 20 images from different areas of each sample were captured at each time point and a minimum of 25 GUVs per sample were used for analysis, per experiment.

The analysis was done using Zen 3.4 software (Zeiss, Germany). The green channel images were used to determine the location of the vesicles. Two circles, $V_{o}$ and $V_{i}$, were drawn at the outer and inner borders of each vesicle, to isolate the signal from the membrane. Similarly-sized circles $B_{o}$ and $B_{i}$ were used to measure the background fluorescence intensity. The mean fluorescence intensity (*I*) and the area (*A*) of the isolated region were analyzed by the software. The fluorescence intensity was then calculated as

$$I_{V}=\frac{A_{V_{o}}I_{V_{o}}-A_{V_{i}}I_{V_{i}}}{A_{V_{o}}-A_{V_{i}}},I_{B}=\frac{A_{B_{o}}I_{B_{o}}-A_{B_{i}}I_{B_{i}}}{A_{B_{o}}-A_{B_{i}}},I_{M}=I_{V}-I_{B},$$

where $I_{V}$ and $I_{B}$ represent the red fluorescence intensity of the vesicle membrane and background, respectively, and $I_{M}$ represents the background-subtracted fluorescence intensity of the GUV membrane. Only when $I_{M}$ for a GUV was greater than 1, the vesicle was considered as a GUV showing fusion. The fusion level (%) was determined by calculating the ratio of GUVs showing fusion to the total number of GUVs with at least 5 μm in diameter in the sample.

### 2.2. Computational Approach

In order to reproduce and predict experimentally observed phenomena, the mathematical model needs to account for three major physical factors: (i) phase separation, (ii) surface density flow, and (iii) electrostatic forces. The thermodynamically consistent NSCH model introduced in [17], and validated against experimental data in [15], accounts only for (i) and (ii), i.e., only phase separation and flow phenomena occurring in lipid membranes can be modeled computationally. In this paper, we extend the NSCH model to include the electrostatic forces between the positively charged lipids in the SUVs and the GUVs, whose average measured zeta potential is negative.

In order to state the model, let Γ be a sphere representing an SUV with a 120 nm diameter and let $c_{i}$ be a fraction of elementary surface area occupied by phase *i*, with $i=L_{o},L_{d}$. We choose $c=c_{L_{o}}$, c∈[0,1], as the representative surface fraction. Let $\rho_{L_{o}}$ and $\rho_{L_{d}}$ be the densities and $\eta_{L_{o}}$ and $\eta_{L_{d}}$ the dynamic viscosities of the two phases. Then, the density and viscosity of the mixture can be written as $\rho =\rho \left(c\right)=\rho_{L_{o}}c+\rho_{L_{d}}\left(1-c\right)$ and $\eta =\eta \left(c\right)=\eta_{L_{o}}c+\eta_{L_{d}}\left(1-c\right)$, respectively. Let u be the area-averaged tangential velocity in the mixture, *p* the thermodynamic interfacial pressure, and μ the chemical potential. Finally, let $F_{e}$ denote the electrostatic force per unit surface area acting on the SUV. The NSCH system with electrostatic forcing that governs the evolution of *c*, u, *p*, and μ in time *t* and space $x\in \Gamma \subset R^{3}$ is given by

(1) $$\begin{array}{c} \;\underset{\mathrm{inertia}}{\underset{︸}{\rho (\partial_{t}u+\left(\nabla_{\Gamma }u\right)u)}}-\underset{\mathrm{lateral}\;\mathrm{stresses}}{\underset{︸}{{\mathrm{div}}_{\Gamma }\left(2\eta E_{s}\left(u\right)\right)+\nabla_{\Gamma }p}}=F_{e}\underset{\mathrm{line}\;\mathrm{tension}}{\underset{︸}{-\sigma_{\gamma }\epsilon^{2}{\;\mathrm{div}}_{\Gamma }\left(\nabla_{\Gamma }c⊗\nabla_{\Gamma }c\right)}}\\ +\underset{\mathrm{chemical}\;\mathrm{momentum}\;\mathrm{flux}}{\underset{︸}{M\theta \left(\nabla_{\Gamma }\left(\theta u\right)\;\right)\nabla_{\Gamma }\mu }} \end{array}$$

(2) $$\;\underset{\mathrm{membrane}\;\mathrm{inextensibility}}{\underset{︸}{{\;\mathrm{div}}_{\Gamma }u=0}}$$

(3) $$\begin{array}{c} \underset{\mathrm{transport}\;\mathrm{of}\;\mathrm{phases}}{\underset{︸}{\partial_{t}c+{\;\mathrm{div}}_{\Gamma }\left(cu\right)}}-\underset{\mathrm{phase}\;\mathrm{masses}\;\mathrm{exchange}\;\;\mathrm{Fick}’s\;\mathrm{law}}{\underset{︸}{{\;\mathrm{div}}_{\Gamma }\left(M\nabla_{\Gamma }\mu \right)}}=0,\;\mu =\underset{\mathrm{mixture}\;\mathrm{free}\;\mathrm{energy}\;\mathrm{variation}}{\underset{︸}{f_{0}^{\prime }\left(c\right)-\epsilon^{2}\Delta_{\Gamma }c}} \end{array}$$

on Γ for $t\in (0,t^{\mathrm{final}}]$. In Equations (1)–(3), $\nabla_{\Gamma }$ stands for the tangential gradient, $\Delta_{\Gamma }$ for the Laplace–Beltrami operator, $E_{s}\left(u\right)=\frac{1}{2}\left(\nabla_{\Gamma }u+{\left(\nabla_{\Gamma }u\right)}^{T}\right)$ is the Boussinesq–Scriven strain-rate tensor, and ${\;\mathrm{div}}_{\Gamma }$ is the surface divergence. Equation (3) provides the definition of the chemical potential, with $f_{0}\left(c\right)=\frac{1}{4}\;c^{2}\;{\left(1-c\right)}^{2}$ being the double-well thermodynamic potential and parameter ϵ>0 representing the width of the (diffuse) interface between the phases. In addition, $\sigma_{\gamma }$ is the line tension coefficient, *M* is the mobility coefficient (see [18]), and $\theta^{2}=\frac{d\rho }{dc}$. Problem (1)–(3) models the total exchange of matter between phases (Equation (3)) with the surface flow described in terms of momentum conservation (Equation (1)) and area preservation (Equation (2)).

To set viscosity and line tension, we referred to experimental work from [19,20,21,22]. In [15], we calculated the value of density for each phase using the estimated molecular weight and molecular surface area for the corresponding phase. However, those values do not take into account the fact that the vesicle is loaded with and surrounded by an aqueous solution. Hence, in this paper we have increased the values to account for the “added mass” coming from such solutions. Table 2 reports the domain ($L_{o}$ phase) area fraction $a_{D}$ and the values or range of values for viscosity, line tension, and density for the compositions under consideration. We note that temperature does not appear in Equations (1)–(3), which describe the evolution of phases and coupled surface flow independently of what initiates phase separation. Indeed, the same model could be used if phase separation was triggered by, e.g., pH [23] instead of temperature. This assumes that variations of the temperature are small (thermodynamically insignificant) after the phase separation is initiated.

Like in our previous works [14,15], we consider degenerate mobility M=Dc(1−c). Parameter *D* is related to thermodynamics properties of matter, just like parameter ϵ in Equation (3), which is the width of the transition layer between ordered and disordered phases. Since the direct evaluation of both *D* and ϵ is not straightforward, in [14] we relied on a data driven approach for their estimation. Our estimate for *D* is $10^{-5}{\left(\mathrm{cm}\right)}^{2}$ s${}^{-1}$, while we found that ϵ= 1 nm is a good estimation for ϵ.

In the simulations, we exposed one SUV to one GUV. Because the GUVs are significantly larger than the SUVs, the curvature of a GUV is negligible at the scale given by the size of an SUV. Hence, we will approximate a GUV with a plane for the computation of the electrostatic force $F_{e}$. Therefore, the electric field E generated by a GUV can be (locally) computed by

(4) $$E=\frac{\sigma }{2\varepsilon_{0}},$$

where σ is the GUV surface charge density and $\varepsilon_{0}$ is the vacuum permittivity ($8.85\;\cdot \;10^{-12}$ F/m). The value of σ is estimated from a linear approximation of Grahame’s formula [24], which is valid in low-potential situations:

(5) $$\sigma \approx \varepsilon \;\cdot \;\varepsilon_{0}\;\cdot \;\kappa \;\cdot \;\Psi_{0},\;\Psi_{0}=\frac{\zeta }{\exp(-\kappa \;\cdot \;x)},$$

where ε is the relative permittivity of water (about 80 at 20 °C), κ is the Debye length parameter for a NaCl solution (10/7 ${\mathrm{nm}}^{-1}$ [25]), $\Psi_{0}$ is the surface potential [25], *x* is the slip plane ( 0.24 nm [25]), and ζ is the zeta potential. The measured average zeta potentials for the GUVs and SUVs are reported in Table 3. It should be noted that for these zeta potential measurements, GUVs were in a sucrose solution and SUVs (PAT1-3) were in a dilute PBS solution. In the absence of ions in the GUV sample, the zeta potential value is higher than that in the presence of ions (e.g., in dilute PBS—see Figure S1B). The negative value for the GUVs is in line with other studies [25,26] and is presumably due to the dipole rearrangement in PC headgroups [26,27].

**Figure 2:**
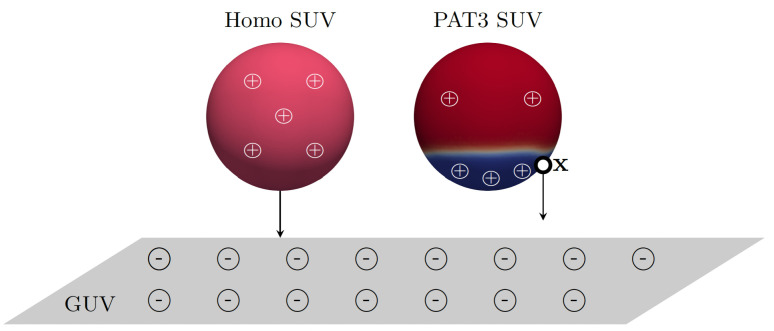
Relative positions of GUV, represented as a plane, and a positively charged SUV, homogeneous (sphere on the left) or phase-separated PAT3 SUV (sphere on the right), in a simulation. The $L_{o}$ phase in the phase-separated SUV is colored in red, while the $L_{d}$ phase is blue.

Once the electric field E is computed, the electrostatic force $F_{e}$ in (1) is given by $F_{e}\left(x\right)=Eq\left(x\right)$, where *q* is a point charge located at x on an SUV (see Figure 2). Since we cannot measure a point charge on an SUV, we resort to an approximation. We find the surface charge density (5) for an SUV using the measured zeta potentials reported in Table 3 for each composition under consideration. With the SUV surface charge density, we obtain the total attraction force density and we distribute it proportionally to the SUV surface. To exemplify the calculation, we consider a PAT3 SUV, which has $a_{D}=70.37\%$, i.e., about 70% of the surface of the SUV is covered by the $L_{o}$ phase (red in Figure 2). For composition PAT3, the concentration of DOTAP in the $L_{d}$ phase (blue in Figure 2) is 41.8% (see Table 4), corresponding to 67.15% of the total DOTAP in the SUV. So, we uniformly distribute 67.15% of the total charge density, and hence force, to the $L_{d}$ phase.

Problem (1)–(3) needs to be supplemented with initial values of velocity $u_{0}$ and state $c_{0}$. We take $u_{0}=0$ (surface fluid at rest) and $c_{0}$ corresponding to a homogeneous mixture. We define $c_{0}$ as a realization of Bernoulli random variable $c_{\mathrm{rand}}$∼$\mathrm{Bernoulli}(a_{D})$ with mean value domain area fraction $a_{D}$. The value of $a_{D}$ is set according to the thermodynamic principles described in [14], which align the values to the measured quantities reported in Table 1.

In generic settings, the solutions to the NSCH problem can only be found numerically. Our numerical scheme for problem (1)–(3) relies on an unfitted finite element method called Trace FEM and an adaptive time-stepping technique [28]. A thorough description of our methodology can be found in [15], with more details available in [17,29]. We performed a mesh refinement study to identify a mesh that yields approximations of u, *p*, *c*, and μ (denoted with $u_{h}$, $p_{h}$, $c_{h}$, $\mu_{h}$) with a satisfying level of accuracy. For the results in Section 3, we adopted a mesh with 225,822 active degrees of freedom (193,086 for $u_{h}$ and 10,912 for $p_{h}$, $c_{h}$, and $\mu_{h}$). The time step Δt adaptively varies from Δt= 4$\;\cdot \;10^{-6}$ s during the fast initial phase of spinodal decomposition to about Δt= 8$\;\cdot \;10^{-4}$ s during the later slow phase of lipid domain coarsening and growth, and up to Δt=4 s when the process is close to equilibrium.

We recall that our numerical method produces numerical solutions that satisfy the mass conservation principle behind (1)–(3):

(6) $$\int_{\Gamma }c_{h}\left(x,t_{n}\right)\;ds=\int_{\Gamma }c_{h}\left(x,t_{n-1}\right)\;ds\;\mathrm{implying}\;\frac{\int_{\Gamma }c_{h}\left(x,t_{n}\right)\;ds}{\int_{\Gamma }1\;ds}≃a_{D},$$

for all n=1,…,N.

## 3. Results and Discussion

In order to investigate the effect of the surface density of cationic lipid DOTAP on liposomes’ fusogenicity, we selected a phase-separating lipid composition DOPC:DPPC:Chol and focused on three different molar ratios reported in Table 1 with distinct domain ($L_{o}$) area fractions $a_{D}$ listed in Table 2. We replaced 15 mol% of DOPC in these liposomal formulations with DOTAP. Given that DOTAP’s acyl-chain chemistry is similar to that of DOPC, we assumed that this lipid would have similar phase partitioning behavior as DOPC and would mostly partition into the $L_{d}$ phase. Table 4 summarizes the lipid distribution among $L_{o}$ and $L_{d}$ phases. These lipid distributions are estimated based on the tie-lines available in the literature [30] and as described in our previous studies [14,15]. With the same DOTAP content, composition PAT3 is expected to have the highest surface density of DOTAP in $L_{d}$ phase because it has the largest $a_{D}$, and composition PAT1 is expected to have the lowest density of DOTAP in its $L_{d}$ phase because it has the smallest $a_{D}$.

**Figure 3:**
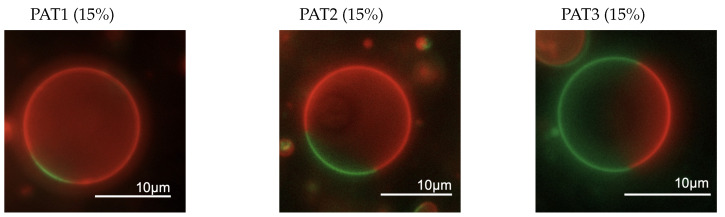
Fluorescence microscopy images of representative phase-separated GUVs with composition PAT1 (**left**), PAT2 (**center**), and PAT3 (**right**) at temperature range of 15–20 °C. Red fluorescence shows $L_{d}$ phase and green fluorescence shows $L_{o}$ phase.

To confirm that the partial replacement of DOPC with DOTAP does not interfere with phase separation in the examined lipid compositions, we first prepared GUVs of these formulations because these micron-sized liposomes can be visualized under optical microscopy. Figure 3 depicts epifluorescent images of representative GUVs with lipid compositions tested here, where the red patches are $L_{d}$ phase and the green patches are $L_{o}$ phase. These images confirmed that the membrane phase separation occurred as expected in all three examined compositions. The results were in good agreement with our previous findings reported in [31].

Next, we prepared SUVs with the above-mentioned compositions to study their fusogenicity. These phase-separating liposomes were compared to homogenous liposomes composed of DOPC with different amounts of DOTAP. We evaluated these SUVs for size and zeta potential. Dynamic light scattering measurements showed that the size distribution of SUVs had a reduction when DOTAP was included in the formulation and was comparable among the three DOTAP-containing phase-separating compositions (PAT1, PAT2, and PAT3) (Figure S1A). The zeta potential values in homogeneous SUVs increased with an increase in their DOTAP content and were similar in all three phase-separating compositions. Interestingly, phase-separating SUVs showed slightly higher zeta potential compared to homogenous SUVs with same DOTAP content (Figure S1B), presumably due to the asymmetrical charge distribution on these SUVs that has been reported to affect the zeta potential values [31,32].

**Figure 4:**
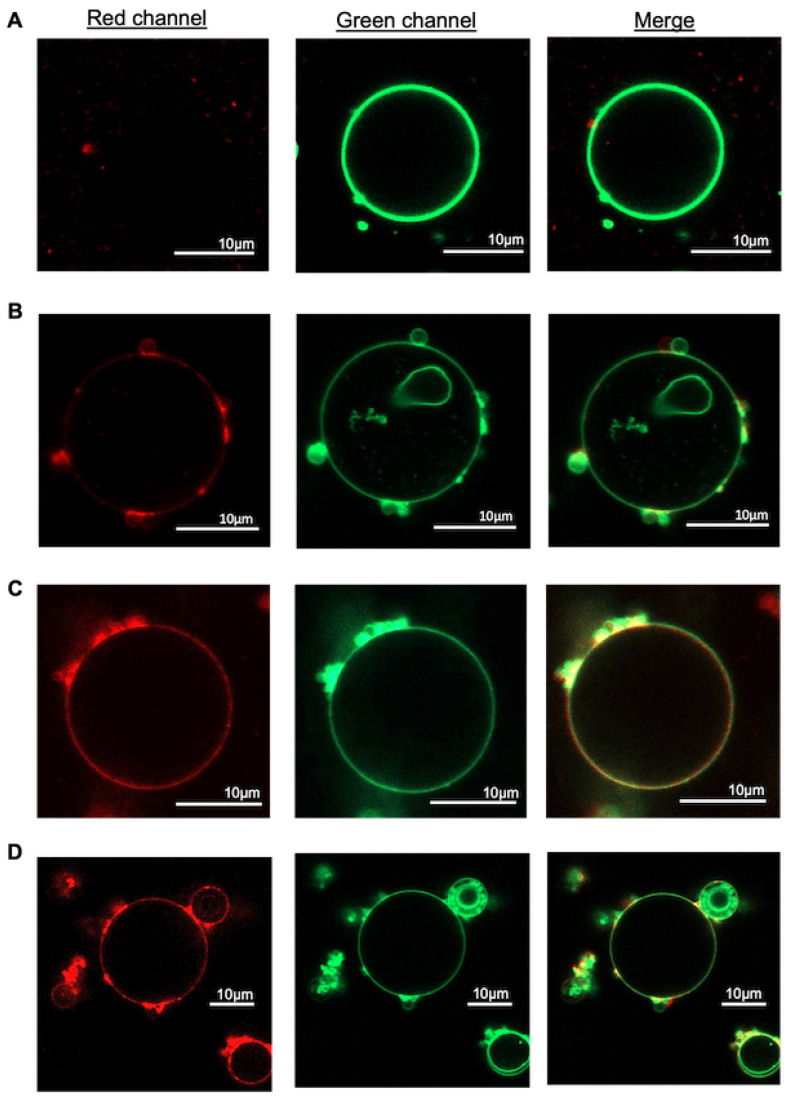
Fluorescence confocal images of representative GUVs composed of DOPC labeled with 0.3% green fluorescent AF488-PE after 10 min incubation with SUVs of (**A**) Homo (0% DOTAP), (**B**) Homo (15% DOTAP), (**C**) Homo (30% DOTAP), (**D**) PAT3 (15% DOTAP).

To examine the ability of DOTAP-containing phase-separating SUVs to fuse into other membranes, we incubated them with GUVs of DOPC composition at 37 °C for 10 min. After the incubation, samples were imaged with confocal microscopy to evaluate the level of fusion of SUVs (labeled with red fluorescence) into GUVs (labeled with green fluorescence). In case of homogeneous SUVs with no DOTAP, the GUVs exhibited only green fluorescence, indicating no significant fusion (Figure 4A). Increasing DOTAP concentration to 15% in homogeneous SUVs resulted in a mixture of both red and green fluorescence on GUVs, suggesting some level of fusion (Figure 4B). Further increasing DOTAP to 30% led to a stronger red fluorescence signal, indicating higher level of fusion (Figure 4C). Interestingly, incubation of GUVs with phase-separating SUVs of PAT3 composition (with 15% DOTAP), led to a much stronger red fluorescence signal in GUV membranes compared to that in case of homogeneous liposomes with 15% DOTAP, and was comparable to that of homogeneous SUVs with 30% DOTAP. It should be noted that the miscibility transition temperature for compositions PAT1 and PAT2 are lower than 37 °C [33] and thus, at this temperature, these membranes are expected to be homogeneous. However, a study by Veatch and her group reported that adhesion between bilayers can result in lipid phase separation at temperatures well beyond the miscibility transition temperature of multicomponent membranes [34]. Given the nature of the fusion process, during which bilayers come in close proximity and adhere to each other, we here assume that all three compositions of PAT1, PAT2, and PAT3 are phase-separated at 37 °C that was used for fusion experiments. This effect has indeed been previously reported in a membrane fusion study [35].

**Figure 5:**
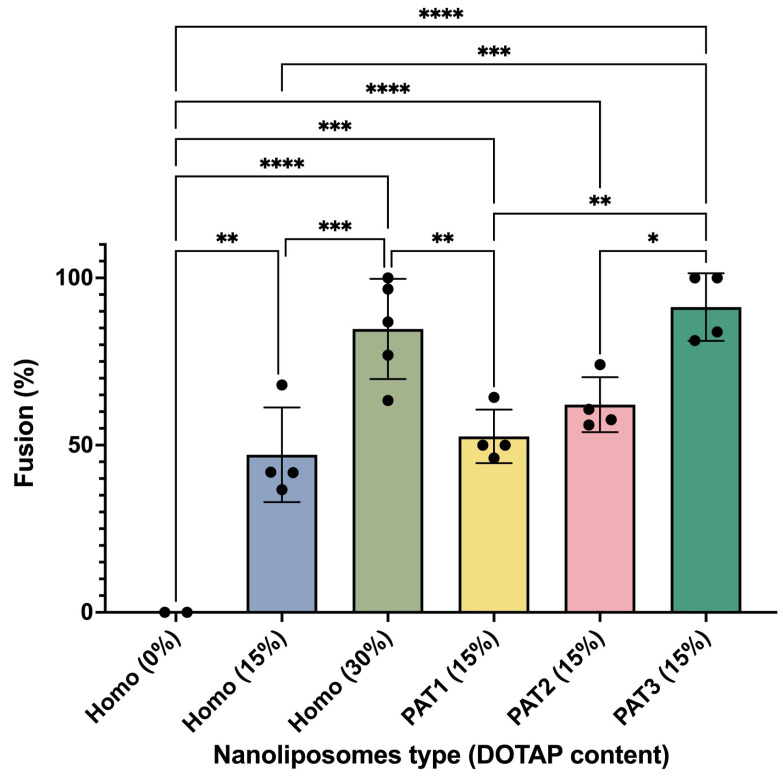
Fraction of GUVs that showed fusion after 10 min incubation with SUVs of different lipid compositions. Data points represent the fraction of GUVs from independent experiments, where in each experiment at least 25 liposomes from randomly selected regions of the sample were used for analysis. Error bars correspond to the standard deviation. Data were statistically analyzed using one-way ANOVA and *: *p* value < 0.05, **: *p* value < 0.01, ***: *p* value < 0.001, ****: *p* value < 0.0001.

To quantify the level of fusion in these experiments, we measured the fraction of GUVs that showed fusion upon incubation with SUVs. As summarized in Figure 5, a higher DOTAP concentration resulted in a higher level of fusion and PAT3 composition, with highest DOTAP density in $L_{d}$ phase, which showed the highest level of fusion. These results showed that increasing the surface density of DOTAP on SUVs even locally (through phase separation) can enhance their fusogenicity.

**Figure 6:**
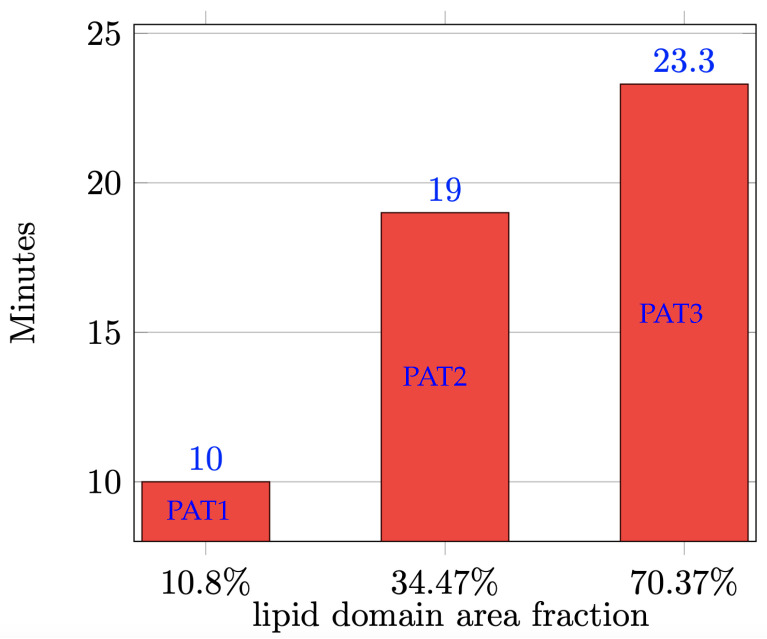
Average time needed for a simulated SUV to reach the equilibrium state (i.e., one patch of the minority phase against the background of the majority phase) for the three compositions under consideration.

Next, we present the computational data and show how they corroborate the observations made from the experiments. As mentioned in Section 2.2, in phase-separated SUVs with cationic lipids there is a complex interplay of the forces driving phase separation, forces driving surface flow, and electrostatic forces. In order to facilitate our understanding of how patches of fusogenic lipids promote fusion, we let the SUVs undergo phase-separation before exposing them to the target model membranes both in the simulations and in the experiments. This serves the purpose of disentangling the effect of phase separation forces from the effect of electrostatic forces. By the time the SUVs are exposed to the model membranes (i.e., >60 min after formation), most SUVs have reached the equilibrium phase-separated state, mostly with one patch of the minority phase against the background of the majority phase. From our previous work [15], we know that membranes of different lipid compositions take different times to reach the equilibrium state, specifically it happens faster for compositions with smaller $L_{o}$ domain area fractions. See Figure 6 for the average time needed to reach the equilibrium for the three lipid compositions under consideration. The average is taken over five simulations with the given composition and random initial distributions (as explained in Section 2.2). We see that a PAT3 SUV ($a_{D}\approx 70\%$) takes more than the double of the time a PAT1 SUV ($a_{D}\approx 11\%$) needs to reach the equilibrium state. We remark that the time in the simulations correspond to physical time.

**Figure 7:**
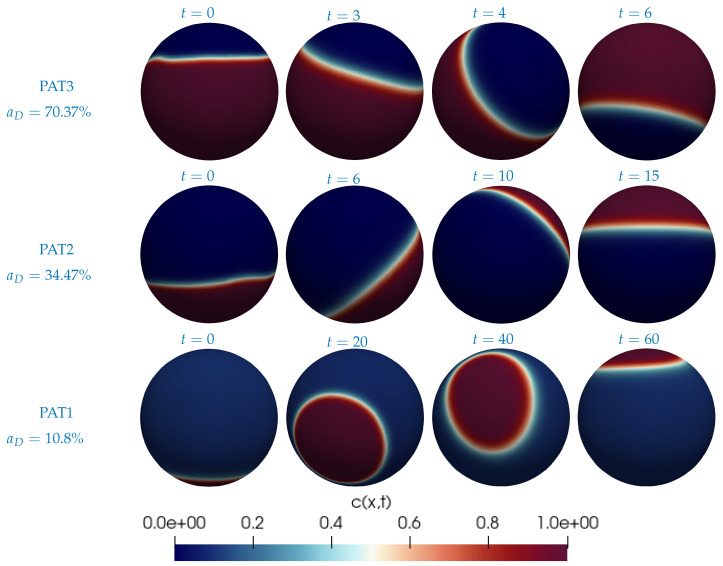
Snapshots of a simulation with the phase-separated PAT3 SUV (top), PAT2 SUV (center), and PAT1 SUV (bottom) at different times (min). Red corresponds to the $L_{o}$ phase and blue to the $L_{d}$ phase. For each composition, the $L_{d}$ phase is initially placed at the top of the SUV (first column). The model membrane, not seen in the figure, is represented as a horizontal plane below the SUV. Click any picture above to run the corresponding full animation.

**Figure 8:**
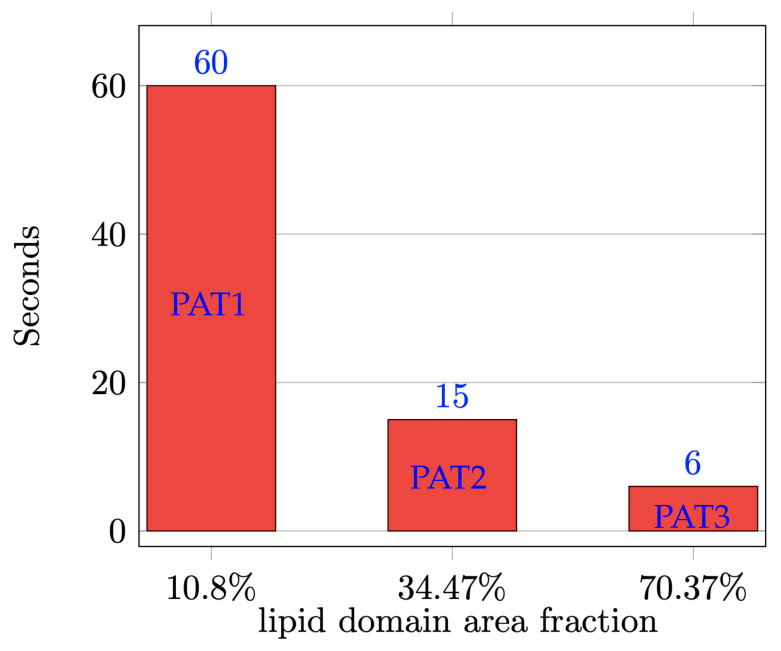
Average time needed to have the $L_{d}$ phase in a simulated SUV face the target model membrane starting from the worst-case scenario configuration.

Once a SUV has reached the phase-separated equilibrium, it is exposed to the target model membrane (equivalent of GUV in experiments), which is represented as a horizontal plane below the SUV in the simulations. Initially, we place the $L_{d}$ phase, which is the phase with the majority of the positive charge, opposite to the model membrane, i.e., at the top of the SUV. See the first column in Figure 7. In a sense, this is the worst-case scenario as it will take the longest to reorient the $L_{d}$ phase so that it faces the model membrane. Once the $L_{d}$ phase faces the model membrane, the SUV is in the optimal configuration to initiate fusion since the majority of the fusogenic lipids are in the $L_{d}$ phase (see Table 4). Figure 7 shows snapshots of the simulated reorientation process for the three compositions. From Figure 7, we clearly see that each SUV takes a different amount of time to have the $L_{d}$ phase face the model membrane. Figure 8 reports such (average) times for each composition. The average is computed again over five simulations per composition, as explained above. We take this time as a proxy for the promotion of fusion since it is the time needed to have the SUV in the optimal configuration for fusion, i.e., with the majority of the fusogenic lipids facing the GUV. Figure 8 informs us that on average a PAT1 SUV takes ten times longer than a PAT3 SUV to reorient its $L_{d}$ phase. Recall that the data used for Figure 5 were acquired after 10 min of incubation. In that amount of time, the simulations predict that all PAT3 SUVs were in the optimal configuration for fusion, regardless of the initial position of the $L_{d}$ phase with respect to the GUV. In contrast, the PAT1 and PAT2 SUVs exposed to a GUV in the worst-case scenario (i.e., $L_{d}$ phase opposite to the GUV) did not have sufficient time to have the $L_{d}$ phase face the GUV. This provides an explanation why the PAT3 SUVs outperform both the PAT1 and PAT2 SUVs. 

## 4. Conclusions

Fluorescence microscopy studies of DOTAP-enriched SUVs fusing with GUVs having a DOPC membrane reveal two main observations: (i) The addition of DOTAP enhances fusogenicity of SUVs, and the fusogenicity levels increase with higher percentages of DOTAP in the SUV composition, (ii) DOTAP-charged SUVs with a phase-separated membrane exhibit higher fusogenicity levels compared to DOTAP-charged SUVs with a homogeneous membrane. Notably, stronger fusogenicity was observed for SUVs with higher concentrations of DOTAP in smaller patches of the membrane in the liquid disordered phase.

While the first observation was expected due to the positive charge carried by DOTAP and the (slightly) negative potential measured for the DOPC GUVs, the second observation is more intriguing. We propose the following explanation for (ii): the phase separation leads to a higher local positive charge density on the SUV membrane, enhancing its interactions with the target GUV membrane. Moreover, the formation of patches and favorable orientation occurs more rapidly for lipid compositions with an area fraction of the liquid disordered phase. Computational results using a state-of-the-art continuum-based model of the two-phased fluid membrane support this suggestion. Findings of this study can be applied for the design of highly fusogenic delivery cationic liposomes with minimal levels of toxicities.

## Figures and Tables

**Table 1:** Lipid composition for the examined liposomes.

Composition	DOPC	DPPC	Chol	Rho-PE
Homo	99.4%	0%	0%	0.6%
PAT1	59.4%	20%	20%	0.6%
PAT2	41.9%	42.5%	15%	0.6%
PAT3	24.4%	50%	25%	0.6%

**Table 2:** Domain ($L_{o}$ phase) area fraction $a_{D}$ (at the given temperature), value or range of values for the density of liquid ordered ($\rho_{L_{o}}$) and liquid disordered ($\rho_{L_{d}}$) phases in Kg/(mol·Å${}^{2}$), viscosity of liquid ordered ($\eta_{L_{o}}$) and liquid disordered ($\eta_{L_{d}}$) phases in $10^{-8}$ Pa·s·m, and line tension in pN for the three membrane compositions under consideration.

Composition	$a_{D}$	$\rho_{L_{o}}$	$\rho_{L_{d}}$	$\eta_{L_{o}}$	$\eta_{L_{d}}$	$\sigma_{\gamma }$
PAT1	10.8% (15 °C)	1401	1172	0.5–6	0.2–0.4	1.2–1.4
PAT2	34.57% (17.5 °C)	1401	1172	0.43–5.7	0.2–0.4	1.2–1.6
PAT3	70.37% (15 °C)	1435	1172	5–8	0.2–0.4	1.2–1.8

**Table 3:** Measured average zeta potentials for the GUVs composed of DOPC (in sucrose solution) and patchy SUVs (in dilute PBS) used in the experiments.

Vesicle	Zeta Potential
GUV	−8.56 mV
PAT1	18.35 mV
PAT2	18.87 mV
PAT3	20.41 mV

**Table 4:** Lipid distribution among the two phases in the examined phase-separated SUVs.

	$L_{d}$ Phase				$L_{o}$ Phase			
**Composition**	**DOTAP**	**DOPC**	**DPPC**	**Chol**	**DOTAP**	**DOPC**	**DPPC**	**Chol**
PAT1 (15%)	16.67%	49.33%	16%	18%	5.56%	16.44%	43%	35%
PAT2 (15%)	22.91%	41.09%	29%	7%	4.65%	8.35%	61%	26%
PAT3 (15%)	41.80%	26.20%	24%	8%	8.61%	5.39%	57%	29%

## Data Availability

Data will be made available upon reasonable request.

## Supplementary Materials

The following supporting information can be downloaded at: https://www.mdpi.com/article/10.3390/biom13101473/s1, Figure S1: The size distribution and zeta potential of the examined SUVs
