# Supplementary material for: On Fusogenicity of Positively Charged Phased-Separated Lipid Vesicles: Experiments and Computational Simulations
Source: Biomolecules. 2023 Sep 30;13(10):1473. doi: 10.3390/biom13101473 (PMC10605210; doi:10.3390/biom13101473)
Supplement: Supplementary file 1 [file biomolecules-13-01473-s001.zip › biomolecules-2587193-supplementary.pdf]

Supplementary Information

Size distribution and zeta potential of homogenous and phase-separating SUVs

Homogenous SUVs composed of DOPC with different amounts of DOTAP and phase-separating SUVs (compositions PAT1, PAT2 and PAT3) with 15 mol% DOTAP were prepared and evaluated for size distribution and zeta potential using Malvern Zetasizer.

As shown in Figure S1A, dynamic light scattering data revealed that the average size of all examined SUV formulations was within 120-150 nm range. The zeta potential of homogenous SUVs increased with an increase in their DOTAP content, as expected. With the same DOTAP content, phase-separating SUVs had slightly higher zeta potential compared to homogenous SUVs, as summarized in Figure S1B.

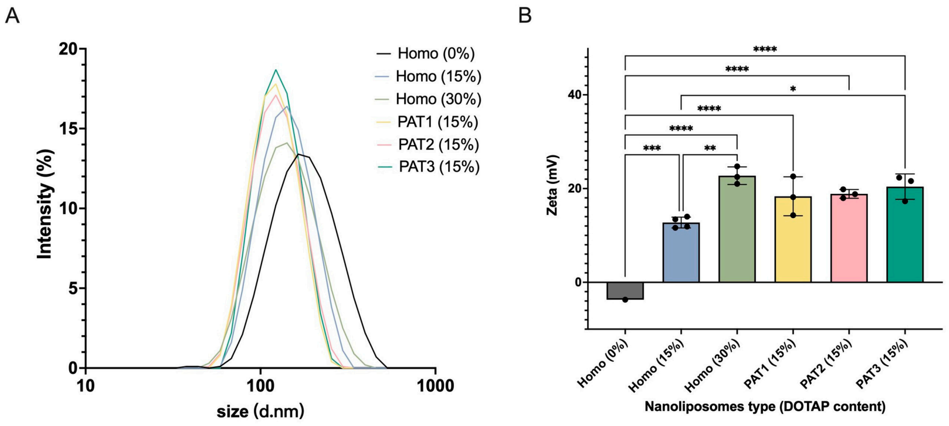

Figure S1: The size distribution (A) and zeta potential (B) of the examined SUVs of different lipid compositions. Percentage of DOTAP is presented in parenthesis. In bar graph, bars represent the average and error bars represent standard deviation with n≥3. Data was statistically analyzed using one-way ANOVA and ****: p values < 0.0001; ***: p values < 0.001; **: p values < 0.01; *: p value < 0.05.
